# Patterns of Screen-Based Media Usage and Associated Health Outcomes Among Rural School Children in Jaipur, Rajasthan: A Cross-Sectional Study

**DOI:** 10.7759/cureus.88947

**Published:** 2025-07-28

**Authors:** Akhileshwar V Reddy, Ashwani Kumar, Khyati M Banker, Kailash C Verma, Dharmendra Mandarwal, Ravindra Manohar, Sumit Ahluwalia, Monank S Patel, Amol Gite, Teja S Pullola

**Affiliations:** 1 Department of Community Medicine, National Institute of Medical Sciences and Research, Jaipur, IND; 2 Department of Obstetrics and Gynaecology, National Institute of Medical Sciences and Research, Jaipur, IND; 3 Department of Internal Medicine, Mamata Academy of Medical Sciences, Hyderabad, IND

**Keywords:** adolescent students, excessive use smartphone, rural india, school children, screen-based media, screen time, smartphone usage

## Abstract

Background

The rapid proliferation of digital devices in rural India has transformed media consumption among adolescents, creating complex patterns of multimodal screen exposure that are influenced by sociodemographic factors. Despite growing concerns about the health and developmental impacts of excessive screen time, limited data exist on rural contexts, where family structures and educational environments differ markedly from those in urban settings.

Objective

This study aims to identify sociodemographic predictors of multimodal screen exposure among rural Indian school children, with a focus on family structure, educational setting, and age.

Methods

A cross-sectional survey was conducted among 370 school children aged 11-17 years in rural Jaipur, Rajasthan. Data were collected using structured questionnaires and media use diaries. Multimodal screen exposure was defined as concurrent use of two or more digital devices. Statistical analyses included descriptive statistics, chi-square tests, ANOVA, and logistic regression.

Results

Joint family structure was associated with 28% lower odds of excessive multimodal screen use compared to nuclear families (OR=0.72, 95% CI: 0.54-0.96, p=0.025). Private school attendance was linked to 2.3 times higher device ownership rates compared to government schools. Age-stratified analysis revealed that older adolescents (15-17 years) were significantly more likely to engage in multimodal screen use than younger adolescents (11-14 years) (41.8% vs. 22.6%, χ²=18.7, p<0.001). Mean daily screen time increased with device multiplicity, from 1.8±0.7 hours for single-device users to 3.1±1.2 hours for multi-device users.

Conclusion

Family structure, school type, and age are key predictors of multimodal screen exposure among rural Indian adolescents. These findings highlight the importance of culturally sensitive, family-centered interventions for screen time management in rural communities.

## Introduction

Screen-based media (SBM) exposure among adolescents has "evolved from single-device interactions to complex multimodal engagement patterns involving simultaneous use of televisions, smartphones, tablets, and gaming consoles" [1]. This transformation is particularly pronounced in developing countries like India, where rapid digital penetration has occurred without corresponding "awareness of healthy usage patterns" [2]. The concept of multimodal screen exposure encompasses not only the duration of screen time but also the complexity of concurrent device usage, which may "amplify the health impacts traditionally associated with single-device use" [3].

Rural populations in India face unique challenges in managing SBM exposure "due to limited access to healthcare guidance, varying socioeconomic conditions, and diverse family structures" [4]. Unlike urban settings where nuclear families predominate, rural areas often maintain traditional joint family systems that may "influence screen time behaviors through shared supervision and resource allocation" [5]. Understanding the sociodemographic predictors of multimodal screen exposure is crucial for developing targeted interventions that acknowledge these cultural and structural differences.

Previous research has demonstrated that "sociodemographic factors significantly influence screen time patterns among children and adolescents" [6]. However, most studies have focused on "urban populations or examined total screen time rather than multimodal usage patterns" [7]. The distinction between single-device and multimodal screen exposure is important because concurrent device usage may lead to different health outcomes, including "increased digital eye strain, attention difficulties, and sleep disturbances" [8].

In rural Indian contexts, factors such as "family structure, school type, parental education, and household size may serve as important predictors of screen exposure patterns" [9]. Joint families, which are prevalent in rural areas, may provide natural supervision mechanisms that limit excessive screen use, while "private schools might introduce different technology exposure patterns compared to government institutions" [10]. Additionally, age and gender differences in screen usage preferences may be more pronounced in traditional rural communities where "gender roles and age-based expectations influence technology access" [11].

This study aims to identify sociodemographic predictors of multimodal screen exposure among rural Indian school children, with particular attention to family structure, educational environment, and developmental factors. By understanding these predictors, healthcare providers and policymakers can develop more effective, culturally appropriate interventions for managing screen time in rural communities.

## Materials and methods

Study design and setting

This cross-sectional analytical study was conducted among school children in the rural catchment area of the Rural Health Training Centre (RHTC) under the Department of Community Medicine at the National Institute of Medical Sciences & Research, Jaipur, Rajasthan, India. The study was conducted over 18 months, spanning the 2022-2025 academic period, and received approval from the National Institute of Medical Sciences and Research (NIMS) University Institutional Ethics Committee (IEC No. EC/NEW/INST/2022/RJ/0118).

Study population and sampling

The target population consisted of students in grades 9-12 from both government and private schools in the study area. Using Cochran's formula with a prevalence of children viewing screens for more than two hours of 68% [12], a sample size of 334 was calculated. Adding 10% for non-response, the final sample size was determined as 370 participants.

A systematic random sampling technique was employed across 10 schools offering senior-secondary education, with 1,459 eligible students forming the sampling frame. Every fourth student (sampling interval K=1459/370≈4) was selected after random selection of the first participant.

Inclusion and exclusion criteria

School children in grades 9-12, students who were present during the study period, and children who provided informed consent, along with permission from school authorities and their parents/guardians, were included in the study. The exclusion criteria comprised children diagnosed with visual problems, cardiovascular diseases, chronic illnesses, or mental illness and children on chronic medications.

Data collection instruments

Sociodemographic Assessment

A structured questionnaire was administered to collect information on age, gender, family structure (nuclear, joint, or third-generation), number of siblings, family size, school type (government/private), and grade level. The questionnaire was available in both English and Hindi to accommodate local language preferences.

SBM Usage Assessment

Screen time patterns were assessed using a comprehensive "media use diary adapted from validated instruments" [13]. The assessment included device availability at home, daily screen time duration on school days and holidays, primary device usage, and concurrent multi-device usage patterns. Specific attention was given to the timing of screen use, including within 30 minutes of waking and bedtime.

Multimodal screen exposure definition

Multimodal screen exposure was defined as the concurrent use of two or more screen-based devices within the same time period, assessed through self-reported usage patterns during the previous week. Participants were categorized into three groups: single-device users (using one device at a time), dual-device users (using two devices concurrently), and multi-device users (using three or more devices simultaneously).

Statistical analysis

Data were analyzed using IBM SPSS Statistics for Windows, Version 23 (Released 2015; IBM Corp., Armonk, New York) and Apple Numbers v11.2 (Apple, Inc., Cupertino, California). Descriptive statistics included frequencies, percentages, means, and standard deviations. Categorical variables were compared using chi-square tests, while continuous variables were analyzed using independent t-tests or Mann-Whitney U tests based on distribution normality. Logistic regression analysis was performed to identify independent predictors of multimodal screen exposure. Statistical significance was set at p<0.05.

Ethical considerations

Written informed consent was obtained from the school principals, and assent was obtained from the participating students. Participants were assured of confidentiality and the right to voluntary participation, with the option to withdraw at any time.

## Results

Sociodemographic characteristics

The study included 370 rural school children with a mean age of 14.29±1.43 years (range: 11-17 years). Gender distribution was balanced with 190 males (51.4%) and 180 females (48.6%). Most participants were enrolled in private schools (n=341, 92.2%), compared to those in government schools (n=29, 7.8%). The largest representation was from the eighth grade (n=141, 38.1%) and the ninth grade (n=139, 37.6%) (Table 1).

**Table 1:** Sociodemographic Characteristics of the Study Participants Data presented as N, %. Statistical significance set at p<0.05.

Characteristic	Category	N	%	95% CI
Age Group	11–14 years	217	58.6	(53.4%, 63.7%)
	15–17 years	153	41.4	(36.3%, 46.6%)
Gender	Male	190	51.4	(46.2%, 56.5%)
	Female	180	48.6	(43.5%, 53.8%)
School Type	Private	341	92.2	(89.5%, 94.9%)
	Government	29	7.8	(5.1%, 10.5%)
Grade Level	8th	141	38.1	(33.3%, 42.9%)
	9th	139	37.6	(32.8%, 42.5%)
	10th	15	4.1	(2.3%, 6.6%)
	11th	70	18.9	(15.0%, 23.4%)
	12th	5	1.4	(0.4%, 3.2%)

Family structure analysis revealed that joint families were predominant (n=232, 62.7%), followed by nuclear families (n=105, 28.4%) and third-generation families (n=33, 8.9%). The mean family size was 11.88±2.97 members, reflecting typical rural Indian household patterns. Regarding siblings, 115 participants (31.1%) had two siblings, 115 (31.1%) had three siblings, and 119 (32.2%) had four or more siblings (Table 2).

**Table 2:** Family Structure and Household Characteristics Data presented as N, % and mean±SD. The chi-square test is used for categorical variables. Statistical significance set at p<0.05.

Characteristic	Category	N	%	Mean±SD	Test Statistic	p-value
Family Type	Nuclear	105	28.4	-	χ²=123.45	<0.001
	Joint	232	62.7	-		
	Third Generation	33	8.9	-		
Family Size	-	-	-	11.88±2.97	-	-
Number of Siblings	0–1	21	5.7	-	χ²=89.23	<0.001
	2	115	31.1	-		
	3	115	31.1	-		
	≥4	119	32.2	-		

Device availability and usage patterns

Device availability was nearly universal, with televisions present in 366 households (98.9%) and smartphones in 315 households (85.1%). Computer/laptop access was reported in 95 households (25.7%), while tablets and gaming consoles were less common at 38 (10.3%) and 23 (6.2%) households, respectively. Despite widespread smartphone availability, only 31 students (8.4%) had personal mobile phone ownership, with 81.4% requiring parental permission before device use (Table 3).

**Table 3:** Device Availability and Usage Patterns by Sociodemographic Factors Data presented as N (%). The chi-square test was used for comparison between school types. Statistical significance set at p<0.05.

Device Type	Overall Availability N (%)	Private School N (%)	Government School N (%)	χ²	p-value
Television	366 (98.9)	337 (98.8)	29 (100.0)	0.35	0.553
Smartphone	315 (85.1)	294 (86.2)	21 (72.4)	4.12	0.042
Computer/Laptop	95 (25.7)	92 (27.0)	3 (10.3)	4.02	0.045
Tablet/iPad	38 (10.3)	37 (10.9)	1 (3.4)	1.56	0.211
Gaming Console	23 (6.2)	23 (6.7)	0 (0.0)	2.09	0.148

Multimodal screen exposure patterns

Analysis of concurrent device usage revealed that 152 participants (41.1%) used single devices, 105 (28.4%) used two devices concurrently, and 113 (30.5%) engaged with three or more devices simultaneously. Mean daily screen time increased progressively with device multiplicity: 1.8±0.7 hours for single-device users, 2.4±0.9 hours for dual-device users, and 3.1±1.2 hours for multi-device users (Table 4).

**Table 4:** Multimodal Screen Exposure by Sociodemographic Characteristics Data presented as N (%). ANOVA is used for continuous variables, while the chi-square test is used for categorical variables. Statistical significance set at p<0.05.

Characteristic	Single Device N (%)	Dual Device N (%)	Multi-Device N (%)	F-statistic	p-value
Age Group				8.45	<0.001
11–14 years	102 (47.0)	66 (30.4)	49 (22.6)		
15–17 years	50 (32.7)	39 (25.5)	64 (41.8)		
Gender				2.87	0.238
Male	75 (39.5)	58 (30.5)	57 (30.0)		
Female	77 (42.8)	47 (26.1)	56 (31.1)		
Family Type				7.23	0.027
Nuclear	36 (34.3)	28 (26.7)	41 (39.0)		
Joint	105 (45.3)	67 (28.9)	60 (25.9)		
Third Generation	11 (33.3)	10 (30.3)	12 (36.4)		
School Type				5.12	0.077
Private	136 (39.9)	98 (28.7)	107 (31.4)		
Government	16 (55.2)	7 (24.1)	6 (20.7)		

Screen time distribution patterns

Screen time patterns differed significantly between school days and holidays. On school days, 319 participants (86.2%) maintained screen time under two hours, while only 51 (13.8%) exceeded this threshold. During holidays, this pattern shifted dramatically, with 138 participants (37.3%) exceeding two hours of screen time. Age-stratified analysis revealed that older adolescents (15-17 years) had significantly higher mean daily screen time (2.13±0.89 hours) compared to younger children (1.87±0.76 hours; t=2.89, p=0.003) (Table 5).

**Table 5:** Screen Time Distribution by Sociodemographic Factors Data presented as N (%) and mean±SD. A paired t-test was used for within-group comparisons. Statistical significance set at p<0.05.

Screen Time Category	School Days N (%)	Holidays N (%)	t-statistic	p-value
Overall Pattern			12.45	<0.001
<2 hours	319 (86.2)	232 (62.7)		
2–4 hours	41 (11.1)	106 (28.7)		
>4 hours	10 (2.7)	23 (6.2)		
No usage	0 (0.0)	9 (2.4)		
By Age Group				
11–14 years (Mean±SD)	1.73±0.68	2.42±1.15	8.23	<0.001
15–17 years (Mean±SD)	1.94±0.82	2.89±1.34	9.12	<0.001
By Family Type				
Nuclear (Mean±SD)	1.92±0.81	2.78±1.28	6.45	<0.001
Joint (Mean±SD)	1.75±0.69	2.51±1.21	8.91	<0.001

Predictors of multimodal screen exposure

Logistic regression analysis identified several significant predictors of multimodal screen exposure (defined as concurrent use of ≥3 devices). Age emerged as the strongest predictor, with each additional year increasing the odds of multimodal exposure by 34% (OR=1.34, 95% CI: 1.18-1.52, p<0.001). Private school attendance was associated with 2.1 times higher odds of multimodal exposure compared to government schools (OR=2.12, 95% CI: 1.15-3.91, p=0.016). Conversely, the joint family structure showed protective effects, with 28% lower odds of multimodal exposure compared to nuclear families (OR=0.72, 95% CI: 0.54-0.96, p=0.025) (Table 6).

**Table 6:** Logistic Regression Analysis of Multimodal Screen Exposure Predictors Multimodal exposure is defined as the concurrent use of three or more devices. Hosmer-Lemeshow test: χ²=6.23, p=0.622. Statistical significance set at p<0.05.

Predictor	β Coefficient	SE	Adjusted OR	95% CI	Wald χ²	p-value
Age (continuous)	0.29	0.07	1.34	(1.18–1.52)	17.23	<0.001
Gender (Male vs. Female)	-0.12	0.18	0.89	(0.62–1.27)	0.44	0.507
School Type (Private vs. Government)	0.75	0.31	2.12	(1.15–3.91)	5.89	0.016
Family Type (Joint vs. Nuclear)	-0.33	0.15	0.72	(0.54–0.96)	4.84	0.025
Family Type (Third Gen vs. Nuclear)	-0.18	0.28	0.84	(0.48–1.46)	0.41	0.521
Number of Siblings (≥3 vs. <3)	0.21	0.16	1.23	(0.90–1.69)	1.72	0.190
(Constant)	-3.87	1.02	0.02	-	14.35	<0.001

Gender- and age-specific patterns

Gender-stratified analysis revealed distinct patterns in multimodal screen exposure. While overall prevalence did not differ significantly between males (30.0%) and females (31.1%), device preferences varied notably. Males showed a higher preference for gaming consoles (68% vs. 23%, χ²=45.2, p<0.001) and computers (42% vs. 28%, χ²=7.8, p=0.005), while females preferred smartphones for social media activities (67% vs. 45%, χ²=18.3, p<0.001). Age-specific analysis demonstrated that the transition from single-device to multimodal usage occurred primarily between the ages of 14 and 15 years, with multimodal exposure prevalence increasing from 22.6% in the 11-14 age group to 41.8% in the 15-17 age group (χ²=18.7, p<0.001).

## Discussion

This study provides novel insights into the sociodemographic predictors of multimodal screen exposure among rural Indian school children, revealing complex relationships between family structure, educational environment, and technology usage patterns. The finding that 30.5% of participants engaged in multimodal screen exposure represents a significant shift from traditional single-device usage patterns, suggesting that rural areas are experiencing rapid digital transformation despite infrastructural challenges. Like the Varadarajan et al. study [14], our findings demonstrate concerning patterns of excessive screen exposure in rural populations.

The protective effect of joint family structures against multimodal screen exposure (OR=0.72) is particularly noteworthy and aligns with the concept of natural supervision mechanisms inherent in traditional Indian family systems. Similar to Khanna et al. [15], our research highlights the importance of family dynamics in adolescent behavior patterns. Joint families typically involve multiple adults who can monitor children's activities, establish shared rules about technology use, and provide alternative social interactions that may reduce dependency on screen-based entertainment. Like the Nandhini et al. study [16], this finding contrasts with urban studies where nuclear families often demonstrate better control over children's screen time, highlighting the importance of cultural context in understanding technology adoption patterns. As demonstrated by Saikia et al. [17], regional variations in family structures significantly influence adolescent behavioral outcomes.

The strong association between private school attendance and multimodal screen exposure (OR=2.12) reflects broader socioeconomic disparities in technology access and educational approaches. Private schools in rural India often emphasize technology integration and may normalize multi-device usage through educational activities. Similar to the findings by Bharati et al. [18], educational environments play crucial roles in shaping technology exposure patterns. Additionally, families choosing private education typically have higher disposable incomes, enabling greater device ownership and less restrictive access policies. Like the Rani et al. study [19], our findings suggest that socioeconomic factors significantly influence technology access and usage patterns. This finding has important implications for targeted interventions, suggesting that technology literacy programs should be differentiated based on educational settings.

The age-related increase in multimodal exposure, with each additional year increasing odds by 34%, reflects developmental patterns in technology adoption and autonomy-seeking behaviors typical of adolescence. As shown by Adelantado-Renau et al. [20], developmental factors significantly influence screen media consumption patterns. The critical transition period, identified between ages 14 and 15 years, aligns with neurobiological changes that affect risk-taking behaviors and susceptibility to peer influence. Similar to Chen et al. [21], our study identifies critical developmental windows for intervention. This timing suggests optimal intervention windows for establishing healthy screen habits before multimodal patterns become entrenched.

Gender differences in device preferences, rather than overall exposure levels, highlight the importance of considering content and context alongside duration in screen time research. Like the Alshoaibi et al. study [22], our findings reveal significant gender-based variations in device usage patterns. The male preference for gaming and computer use versus the female preference for smartphone-based social media activities reflects broader societal patterns and may have different health implications requiring gender-specific intervention approaches. As demonstrated by Simon et al. [23], gender-specific approaches to screen time management may be more effective.

The dramatic increase in screen time during holidays (from 13.8% to 37.3%, exceeding two hours) underscores the importance of structured environments in regulating screen use. This pattern suggests that schools inadvertently serve as natural screen time regulators, and interventions should specifically target non-school periods when parental supervision may be reduced. Similar to Mohan et al. [24], our study highlights the impact of environmental structure on screen exposure patterns.

Several limitations should be acknowledged. The cross-sectional design precludes causal inferences about the relationships identified. Self-reported screen time data may be subject to recall bias and social desirability effects, particularly given cultural sensitivities around technology use in rural communities. Like the Zacharia et al. study [25], methodological considerations regarding self-reporting must be acknowledged. The predominance of private school students (92.2%) limits the generalizability of the findings to the broader rural population, where government schools are more common. Additionally, the study did not capture content quality or educational versus recreational usage patterns, which may be important moderators of screen time effects. As noted by Sharma et al. [26], a comprehensive assessment of screen exposure requires consideration of multiple dimensions beyond duration.

Future research should employ longitudinal designs to establish the temporal relationships between sociodemographic factors and multimodal screen exposure development. Objective measurement tools, such as smartphone applications that track actual usage patterns, would enhance accuracy and reduce reporting bias. Similar to methodological approaches recommended by Lovibond et al. [27], standardized assessment tools would improve research reliability. Qualitative studies exploring family decision-making processes around technology use could provide deeper insights into the mechanisms underlying the protective effects of joint family structures. Like the validation approach by Bastien et al. [28], mixed-methods research could provide a more comprehensive understanding.

The findings have important implications for public health interventions and policy development. The protective effects of joint families suggest that family-centered approaches leveraging traditional supervision mechanisms may be more effective than individual-focused interventions in rural contexts. Similar to recommendations by Kar et al. [29], culturally appropriate interventions should leverage existing family structures. School-based programs should be tailored to institutional types, with private schools requiring different approaches than government schools. The identification of the 14-15-year transition period provides a specific target for preventive interventions before problematic patterns become established. As emphasized by the World Health Organization [30], age-appropriate guidelines for screen time management are essential for healthy development.

## Conclusions

This study identifies significant sociodemographic predictors of multimodal screen exposure among rural Indian school children, with age, school type, and family structure emerging as key determinants. The protective effect of joint family structures and the increased risk associated with private school attendance highlight the importance of cultural and socioeconomic factors in technology adoption patterns. The critical transition period between the ages of 14 and 15 years provides an optimal intervention window for establishing healthy screen habits.

These findings support the development of targeted, culturally sensitive interventions that leverage existing family structures and address specific risk groups. Future research should focus on longitudinal studies with objective measurement tools to better understand the temporal relationships and mechanisms underlying these associations. Public health strategies should prioritize family-centered approaches and consider the unique characteristics of rural communities when designing screen time management programs.

## Appendices

Questionnaire

**Table 7:** Sociodemographic Information Section

Section	Question	Response Options	Variable Type	Coding
Basic Demographics	What is your age?	Open-ended (11-17 years)	Continuous	Age in years
	What is your gender?	1. Male 2. Female	Categorical	1=Male, 2=Female
	What grade are you currently in?	1. 8th Grade 2. 9th Grade 3. 10th Grade 4. 11th Grade 5. 12th Grade	Ordinal	1-5
Educational Setting	What type of school do you attend?	1. Government School 2. Private School	Categorical	1=Government, 2=Private
Family Structure	What type of family do you live in?	1. Nuclear Family (parents and children only) 2. Joint Family (grandparents, parents, children) 3. Third Generation Family (great-grandparents included)	Categorical	1=Nuclear, 2=Joint, 3=Third Gen
	How many people live in your household?	Open-ended	Continuous	Number of members
	How many siblings do you have?	1. 0-1 siblings 2. 2 siblings 3. 3 siblings 4. 4 or more siblings	Ordinal	1-4

**Table 8:** Device Availability and Access Assessment

Section	Question	Response Options	Variable Type	Coding
Household Device Availability	Which of the following devices are available in your home? (Check all that apply)	1. Television 2. Smartphone 3. Computer/Laptop 4. Tablet/iPad 5. Gaming Console 6. None of the above	Multiple Response	1=Available, 0=Not Available
Personal Device Ownership	Do you have your own mobile phone?	1. Yes 2. No	Categorical	1=Yes, 0=No
Device Access Control	Do you need to ask permission from parents/guardians before using devices?	1. Always 2. Sometimes 3. Rarely 4. Never	Ordinal	1-4
Primary Device Usage	Which device do you use most frequently?	1. Television 2. Smartphone 3. Computer/Laptop 4. Tablet/iPad 5. Gaming Console	Categorical	1-5

**Table 9:** Screen Time Assessment

Section	Question	Response Options	Variable Type	Coding
Daily Screen Time - School Days	On average, how many hours per day do you spend using screens on school days?	1. Less than 1 hour 2. 1-2 hours 3. 2-3 hours 4. 3-4 hours 5. More than 4 hours 6. No screen use	Ordinal	1-6
Daily Screen Time - Holidays	On average, how many hours per day do you spend using screens during holidays/weekends?	1. Less than 1 hour 2. 1-2 hours 3. 2-3 hours 4. 3-4 hours 5. More than 4 hours 6. No screen use	Ordinal	1-6
Morning Screen Use	Do you use screens within 30 minutes of waking up?	1. Always 2. Often 3. Sometimes 4. Rarely 5. Never	Ordinal	1-5
Bedtime Screen Use	Do you use screens within 30 minutes of bedtime?	1. Always 2. Often 3. Sometimes 4. Rarely 5. Never	Ordinal	1-5

**Table 10:** Multimodal Screen Exposure Assessment

Section	Question	Response Options	Variable Type	Coding
Concurrent Device Usage	How often do you use multiple devices at the same time?	1. Never 2. Rarely 3. Sometimes 4. Often 5. Always	Ordinal	1-5
Multi-Device Patterns	What is the maximum number of devices you typically use simultaneously?	1. One device only 2. Two devices 3. Three devices 4. Four or more devices	Ordinal	1-4
Device Combination Patterns	Which device combinations do you most commonly use together? (Check all that apply)	1. TV + Smartphone 2. Computer + Smartphone 3. TV + Gaming Console 4. Tablet + Smartphone 5. Computer + Gaming Console 6. Other combinations	Multiple Response	1=Used, 0=Not Used
Multi-tasking Activities	When using multiple devices, what activities do you typically engage in? (Check all that apply)	1. Watching TV while texting 2. Gaming while listening to music 3. Studying while checking social media 4. Video calling while browsing 5. Other activities	Multiple Response	1=Yes, 0=No

**Table 11:** Content and Context Assessment

Section	Question	Response Options	Variable Type	Coding
Content Preferences	What type of content do you primarily consume on screens? (Check top 3)	1. Educational videos/content 2. Entertainment/movies 3. Social media 4. Gaming 5. News/information 6. Communication (calls/messages) 7. Music/audio content	Multiple Response	Rank 1-3
Usage Context	Where do you most commonly use screens?	1. Own bedroom 2. Family living area 3. Study room 4. Kitchen/dining area 5. Outdoor spaces 6. Multiple locations	Categorical	1-6
Social Context	Do you typically use screens alone or with others?	1. Always alone 2. Usually alone 3. About equally alone and with others 4. Usually with others 5. Always with others	Ordinal	1-5
Supervision Level	How often are adults present when you use screens?	1. Always 2. Often 3. Sometimes 4. Rarely 5. Never	Ordinal	1-5
